# ALK F1174S mutation impairs ALK kinase activity in EML4-ALK variant 1 and sensitizes EML4-ALK variant 3 to crizotinib

**DOI:** 10.3389/fonc.2023.1281510

**Published:** 2024-01-09

**Authors:** Jikui Guan, Tzu-Po Chuang, Anders Vikström, Ruth H. Palmer, Bengt Hallberg

**Affiliations:** ^1^Institute of Pediatric Medicine, Children’s Hospital Affiliated to Zhengzhou University, Zhengzhou, China; ^2^Department of Medical Biochemistry and Cell Biology, Institute of Biomedicine, Sahlgrenska Academy, University of Gothenburg, Gothenburg, Sweden; ^3^Department of Pulmonary Medicine, Linköping University Hospital, Linköping, Sweden

**Keywords:** anaplastic lymphoma kinase, lung cancer, resistance, neuroblastoma, tyrosine kinase inhibitor, lorlatinib

## Abstract

**Objective:**

To assess the influence of F1174S mutation on kinase activity and drug sensitivity of the echinoderm microtubule-associated protein-like 4 (EML4) and anaplastic lymphoma kinase (ALK) fusion (EML4-ALK) variants 1 and 3.

**Methods:**

We constructed mammalian expression plasmids of both wildtype and F1174 mutant EML4-ALK variants 1 and 3, and then characterized them with cell models by performing immunoblotting, neurite outgrowth assay, focus formation assay as well as protein stability assay. Drug sensitivity to ALK tyrosine kinase inhibitors was also compared between wildtype and F1174 mutant EML4-ALK fusions. In addition, we characterized the effect of different F1174 kinase domain mutations in the context of EML4-ALK fusions.

**Results:**

In contrast to the oncogenic ALK-F1174S mutation that has been reported to be activating in the context of full-length ALK in neuroblastoma, EML4-ALK (F1174S) variant 1 exhibits impaired kinase activity leading to loss of oncogenicity. Furthermore, unlike the previously reported F1174C/L/V mutations, mutation of F1174 to S sensitizes EML4-ALK variants 3a and 3b to crizotinib.

**Conclusion:**

These findings highlight the complexity of drug selection when treating patients harboring resistance mutations and suggest that the F1174S mutation in EML4-ALK variant 1 is likely not a potent oncogenic driver. Additional oncogenic driver or other resistance mechanisms should be considered in the case of EML4-ALK variant 1 with F1174S mutation.

## 1 Introduction

Anaplastic lymphoma kinase (ALK) is a receptor tyrosine kinase (RTK) first identified as an oncogenic fusion protein with nucleophosmin (NPM) in a subset of anaplastic large cell non-Hodgkin’s lymphomas in 1994 (1). Thereafter, aberrant ALK, either as a fusion partner or a full-length protein with activating mutations or overexpression, has been reported in a wide range of human malignancies including non-small cell lung cancer (NSCLC) and neuroblastoma (NB) (2, 3).

In NB, full-length ALK with aberrant activity caused by activating point mutations or gene amplification is observed in 5-10% of primary cases (4–7) and is more frequently found in relapsed NB cases (8, 9). Mutations in three hotspots - F1174, R1275 and F1245 -account for the majority of activating mutations found in ALK-positive NB cases. ALK-driven NB patients usually have a poor prognosis and ALK activity is highly correlated to aggressive tumor progression, metastasis and chemotherapy resistance (10).

Fusion of ALK with echinoderm microtubule-associated protein-like 4 (EML4) has been reported in approximately 5% of non-small cell lung cancer (NSCLC) (11–14). EML4-ALK fusion arises from an inversion on chromosome 2p that joins EML4 to exons 20 to 29 of ALK. The N-terminal EML4 fusion portion contains a coiled-coil domain that is necessary for dimerization or oligomerization of the fusion protein, resulting in constitutive activation of the ALK tyrosine kinase (11). Numerous EML4-ALK variants have been identified, reflecting multiple sites of chromosomal inversion with the most common variants referred to as EML4-ALK variant 1 (V1) and EML4-ALK variant 3a/b (V3a/b) (13–16).

Multiple ATP-competitive ALK tyrosine kinase inhibitors (TKIs) have been developed and approved by FDA to date (14, 16). Despite marked anti-tumor efficacy observed both in preclinical settings and in the clinic, acquired resistance to ALK TKIs inevitably occurs during the course of treatment. Secondary resistance mutations in ALK kinase domain represent one of the major mechanisms underlying ALK TKI resistance. So far, numerous ALK resistance mutations including L1196M, I1171T/N/S, L1152P/R, 1151Tins, F1174C/L/V, C1156Y/T, I1171T/N/S, S1206C/Y, G1269A/S, V1180L, L1198F, E1210K, G1202R and D1203N have been reported in ALK TKI treated patients that have relapsed or in experimentally developed resistant cell line models (3, 16, 17). Moreover, complex mutations, such as co-occurrence of E1210K + S1206C, E1210K + D1203N, L1198F + C1156Y and G1269A + G1202R, have been reported in patients resistant to treatment with the third generation inhibitors brigatinib and lorlatinib (18, 19).

In this study, we characterized a so-far unreported EML4-ALK F1174S mutation identified in a Swedish NSCLC patient. In contrast to the previously described F1174C/L/V resistance mutations, the F1174S mutation sensitized EML4-ALK V3a/b to crizotinib, the first generation ALK TKI FDA approved for ALK-positive NSCLC. Further analysis showed that F1174S mutation results in an instable EML4-ALK V1 protein, leading to a marked loss of ALK kinase activity and oncogenic potential. Taken together, our data indicate that the EML4-ALK F1174S mutation differs from other F1174 mutations in the context of EML4-ALK fusions and highlights the complex behavior of resistant mutations in ALK-positive NSCLC.

## 2 Materials and methods

### 2.1 Cell culture and inhibitors

PC12 cells (ATCC CRL-1721) were cultured in RPMI 1640 medium supplemented with 7% heat inactivated horse serum and 3% non-heat inactivated fetal bovine serum (FBS) and a mixture of 1% penicillin/streptomycin. SK-N-AS cells (ATCC CRL-2137) were cultured in RPMI 1640 medium supplemented with 10% heat inactivated FBS and a mixture of 1% penicillin/streptomycin. NIH 3T3 cells (ATCC CRL-1658) were cultured in DMEM medium supplemented with 10% heat inactivated FBS and a mixture of 1% penicillin/streptomycin. NL20 cells (ATCC CRL-2503) were cultured in Ham’s F12 medium with 1.5 g/L sodium bicarbonate, 2.7 g/L glucose, 2.0 mM L-glutamine, 0.1 mM nonessential amino acids, 0.005 mg/ml insulin, 10 ng/ml epidermal growth factor, 0.001 mg/ml transferrin, 500 ng/ml hydrocortisone and 4% heat inactivated FBS and a mixture of 1% penicillin/streptomycin. DFCI032 (established at Dana Farber Cancer Institute from a treatment naïve female NSCLC patient) and H2228 cells (ATCC CRL-5935) were cultured in RPMI 1640 medium supplemented with 10% heat inactivated FBS and a mixture of 1% penicillin/streptomycin. All cells were cultured under 37°C, 95% humidity and 5% CO_2_ conditions. ALK TKIs, including crizotinib (T1661), lorlatinib (T3061), alectinib (T1936), brigatinib (T3621) and ceritinib (T1791), were purchased from TargetMol Chemicals Inc (Boston, MA).

### 2.2 Generation of human EML4-ALK constructs

EML4-ALK V1, V3a and V3b cDNAs were synthesized by GeneScript (Piscataway, NJ) and cloned into pcDNA3 vector. Mutation of either F1174S or F1174L in all three EML4-ALK variants were subsequently generated by substituting a 1.26 kb long BlpI-BsiWI fragment of the ALK kinase domain with the corresponding part from full length ALK (F1174S) or ALK (F1174L) constructs respectively. The generation of full length ALK (F1174S) and ALK (F1174L) constructs has been described previously (10). EML4-ALK F1174S→WT V1 and EML4-ALK F1174L→F1174S V1 constructs were generated with QuickChange II Site-Directed Mutagenesis Kit (Agilent, Santa Clara, CA), in which the F1174S mutation was either mutated back to wildtype, or F1174L mutation was mutated to F1174S, respectively. All the above variants and mutations were confirmed by sequencing.

### 2.3 Neurite outgrowth assay

PC12 cells (2×10^6^) were transfected by electroporation in an Amaxa electroporator (Lonza, Basel, Switzerland), using 0.5 μg of empty pcDNA3 vector, full length ALK (F1174S) or ALK (F1174L) construct, or various EML4-ALK constructs as indicated together with 0.5 μg of pEGFP-C1 and 100 μL of Ingenio electroporation solution (Mirus Bio LCC, Madison, WI). After electroporation, cells were transferred to RPMI 1640 medium supplemented with 7% horse serum and 3% FBS and seeded into 24-well plates. Two days after transfection, the percentage of GFP-positive and neurite-carrying cells versus GFP-positive cells was calculated using a Zeiss Axiovert 40 CFL microscope. Light microscopy images were taken under Olympus CK40 Inverted Phase Contrast Microscope with DP12 camera. Representative rectangle raw images were cropped to square ones with the same size prior to being used in the figure. To be judged as a neurite-carrying cell, the neurites of the cell had to reach at least twice the length of the cell body. Experiments were performed in triplicate and each sample within an experiment was assayed in duplicate. The significance of neurite outgrowth difference between two groups was calculated with two-tailed paired student’s *t*-test.

### 2.4 Focus formation assay

NIH 3T3 cells (5×10^4^) seeded in collagen-coated 12-well plates were transfected with 1.5 μg of pcDNA3 vector, pcDNA3-EML4-ALK V1, pcDNA3-EML4-ALK V3a, pcDNA3-EML4-ALK V3b, pcDNA3-EML4-ALK (F1174S) V1, pcDNA3-EML4-ALK (F1174S) V3a, pcDNA3-EML4-ALK (F1174S) V3b, pcDNA3-EML4-ALK (F1174L) V1, pcDNA3-EML4-ALK (F1174L) V3a, pcDNA3-EML4-ALK (F1174L) V3b, pcDNA3-ALK (F1174S) or pcDNA3-ALK (F1174L) construct together with 5 μl of Lipofectamine 2000 (Invitrogen, Carlsbad. CA) in 0.3 ml Opti-MEM (Invitrogen, Carlsbad. CA). The next day, 60% of the cells were transferred to six-well plates and maintained in DMEM with 10% FBS and 0.5 mg/ml G418 until the cells reached confluence. Cells were cultivated in DMEM with 5% FBS and 0.25 mg/ml G418 for another 10 days, after which cells were fixed with methanol and stained with 0.25% crystal violet to visualize focus formation.

### 2.5 Reverse transcription-polymerase chain reaction (RT-PCR)

Total RNA was extracted from DFCI032 cells treated with 30 nM lorlatinib or mock for 24 hours with PureLink RNA Mini kit (Invitrogen, Carlsbad. CA). Reverse transcription was performed with the SuperScript IV First-Strand Synthesis System (Invitrogen, Carlsbad. CA) following the manufacturer’s protocol. PCR was performed with DreamTaq Hot Start Green PCR Master Mix (ThermoScientific, Waltham, MA). The PCR primers used for *ALK* were 5’-CGTTGCAACTGGGAGACTTC-3’ and 5’-GAGTGTGCGACCGAGCTCAG-3’; and the primers for *Actin* were 5’-CCTGGAGAAGAGCTACGAGC-3’ and 5’-GTACCACACGGAGTACTTGC-3’.

### 2.6 Cell transfection, treatment, lysis, and immunoblotting

PC12 cells (2×10^6^) were transfected by electroporation with 0.5 μg of wildtype or F1174S mutant EML4-ALK constructs and were cultured with or without 30 nM of lorlatinib for 24 hours. For the time course of lorlatinib inhibition, PC12 cells (2×10^6^) transfected with 0.5 μg of EML4-ALK V1, V3a, or V3b plasmids were cultured with 30 nM lorlatinib for 0, 12, 24 and 48 hours respectively. SK-N-AS cells were transfected with Lipofectamine 3000 (Invitrogen, Carlsbad. CA) following the manufacturer’s instruction. To assay protein stability, NL20 cells transfected with wildtype, F1174S mutant or F1174L mutant EML4-ALK V1, V3a or V3b respectively were treated with 20 μg/ml cycloheximide (CHX) (Merck, Darmstadt, Germany) for 0, 7, 16 or 24 hours. For ALK inhibitor profiling on EML4-ALK V3a, EML4-ALK V3b, EML4-ALK (F1174S) V3a and EML4-ALK (F1174S) V3b, NL20 cells were transfected with X-tremeGENE 9 DNA transfection reagent (Roche, Basel, Switzerland). After 24-36 hours culture, cells were treated with serial dilutions of the indicated inhibitors for 2 hours. Lung cancer cells DFCI032 and H2228 were cultured in the absence or presence of 30 nM lorlatinib for 24 hours prior to total RNA extraction or cell lysis.

Cells were washed with ice-cold PBS prior to harvest in lysis buffer [25 mM of Tris-Cl, pH7.5, 150 mM of NaCl, 1% (v/v) Triton X-100, 1 mM of DTT, and protease inhibitor cocktail]. Cell lysates were clarified by centrifugation at 14,000 rpm for 15 minutes at 4°C. Samples were boiled in 1x SDS sample buffer and analyzed by immunoblotting. Enhanced chemiluminescence (ECL) and fluorescence blot images were taken with LI-COR Odyssey FC instrument and Image Studio 5.2 software (Lincoln, NE). Image processing methods (such as changes to the brightness and/or contrast) were applied to whole blots and the changes did not alter the information illustrated in the figure (for processing details, refer to Supplementary Data). The intensity of different protein bands was quantified with Image Studio Lite 5.2 software (Lincoln, NE). Ratios were shown either directly or normalized. For crizotinib inhibition profiling, data were normalized to the 0 nM inhibitor samples. GraphPad Prism 6.0 was used to calculate IC50 values by fitting data to a log (inhibitor concentration) vs. normalized response (variable slope) equation.

Primary antibodies used for immunoblotting: anti-pan-ERK (1:10,000) mouse monoclonal antibody (mAb) from BD Transduction Laboratories (Franklin Lakes, NJ); anti-pALK (Y1604) rabbit mAb, anti-pERK1/2 (T202/Y204) rabbit mAb, anti-pAKT (S473) rabbit mAb, anti-Actin rabbit mAb, and anti-ALK (31F12) mouse mAb from Cell Signaling Technology (Danvers, MA); anti-β-Tubulin mouse mAb from ThermoScientific (Waltham, MA). Horseradish-peroxidase-conjugated secondary antibodies goat anti-rabbit IgG and goat anti-mouse IgG (1:5,000) were from Thermo Scientific (Waltham, MA). Fluorescent secondary antibodies: goat-anti-rabbit 680RD and goat-anti-mouse 800CW were purchased from LI-COR (Lincoln, NE).

## 3 Results

### 3.1 EML4-ALK variant 1 exhibits decreased stability and signaling output

An EML4-ALK F1174S mutation was identified in a Swedish NSCLC patient who developed resistance after 13 months of crizotinib treatment. The patient was subsequently treated with alectinib for 6 months, followed by ceritinib for 3 months and finally brigatinib for 1 month prior to deceasing, with no detectable response to any of the prescribed ALK TKIs. While the F1174S mutation has not previously been reported in NSCLC, it has been shown to correlate with aggressive tumor progression in a relapsed NB harboring a F1174S mutation in full length ALK (10).

Many EML4-ALK variants have been described, with the most common variants being V1 and V3a/b (14, 16, 20, 21). As no information was available regarding the EML4-ALK variant in this patient, we initially characterized the effect of F1174S mutation in EML4-ALK V1, EML4-ALK V3a and EML4-ALK V3b, since these variants account for more than 80% of ALK-positive NSCLC cases (11, 13, 14, 16, 21). To investigate the kinase activity of both wildtype and F1174S/L mutant EML4-ALK variants, we introduced them into PC12 cells, cells derived from a pheochromocytoma of the rat adrenal medulla, to characterize protein expression, autophosphorylation, activation of known downstream pathways, and ability to induce neurite outgrowth. Sustained ERK1/2 signaling induces neuronal differentiation of PC12 cells, and this induced neurite outgrowth provides an excellent readout of ALK activity in these cells (22–24).

**Figure 1:**
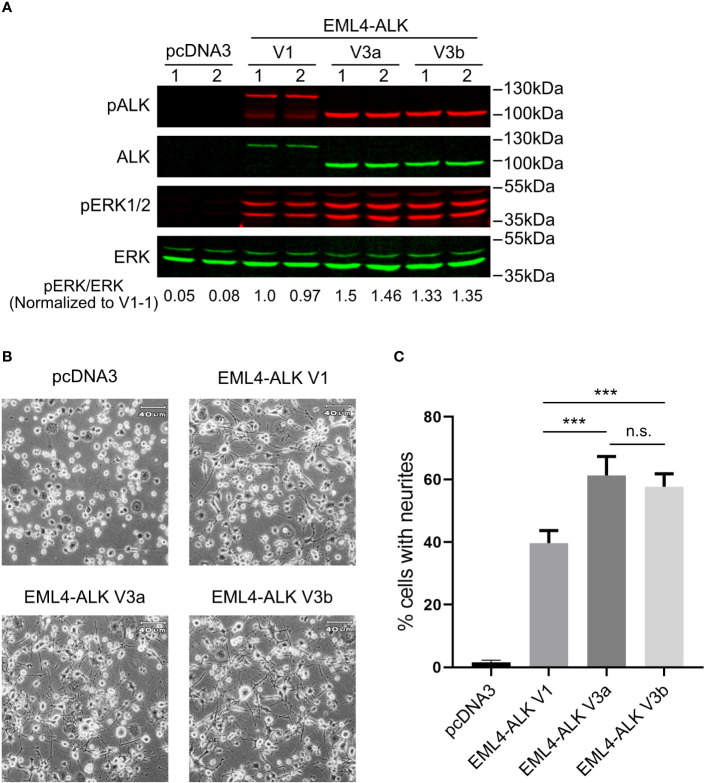
EML4-ALK-V1 exhibits decreased stability and signaling output. **(A)** PC12 cells were transfected with plasmids as indicated in duplicate. Lysates were blotted with phospho-ALK, pan-ALK, phospho-ERK1/2 and pan-ERK antibodies. Phospho-ERK/pan-ERK ratios were normalized to the first EML4-ALK V1 sample. **(B)** Representative light microscope images showing neurite outgrowth of PC12 cells transfected with different EML4-ALK variants as indicated. Scale bar = 40 μm. **(C)** Percentage of transfected PC12 cells carrying neurites. Both EML4-ALK V3a and EML4-ALK V3b induced significantly more neurite outgrowth when compared with EML4-ALK V1 (***p<0.005, n.s. no significance, two-tailed paired student’s *t*-test). Bars represent mean percentage ± STD from three independent experiments.

**Figure 2:**
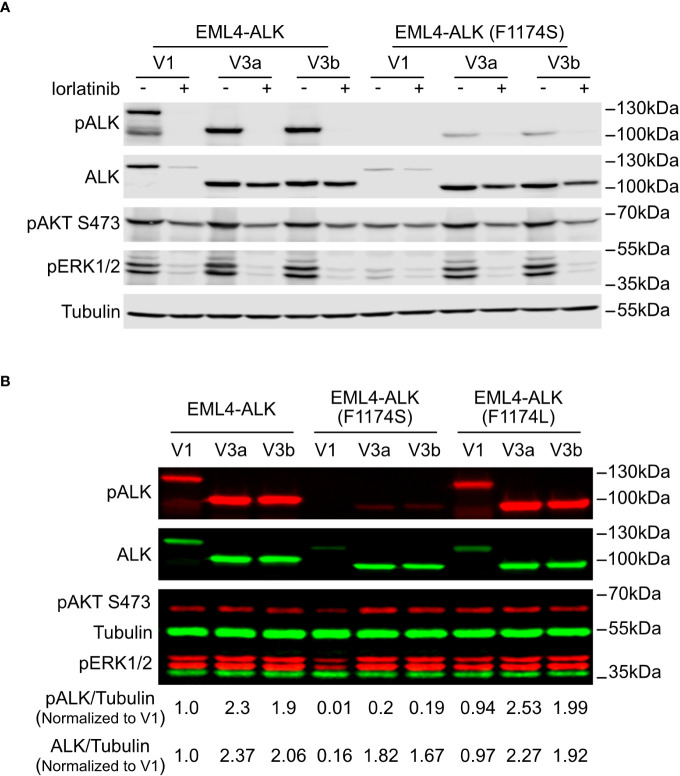
F1174S mutation further impairs stability and signaling output of EML4-ALK V1. **(A)** Immunoblotting analysis of PC12 cells transfected with wildtype or F1174S mutant EML4-ALK variants as indicated. Cells were cultured for 24 hours in the absence or presence of lorlatinib prior to immunoblotting with phospho-ALK, pan-ALK, phospho-AKT and phospho-ERK1/2 antibodies. Tubulin was used as loading control. **(B)** Immunoblotting analysis of SK-N-AS cells transfected with wildtype, F1174S mutant or F1174L mutant EML4-ALK variants as indicated. Lysates were immunoblotted with phospho-ALK, pan-ALK, phospho-AKT and phospho-ERK1/2 antibodies. Tubulin was used as loading control. Phosphor-ALK/Tubulin and pan-ALK/Tubulin ratios were normalized to the wildtype EML4-ALK V1 sample.

**Figure 3:**
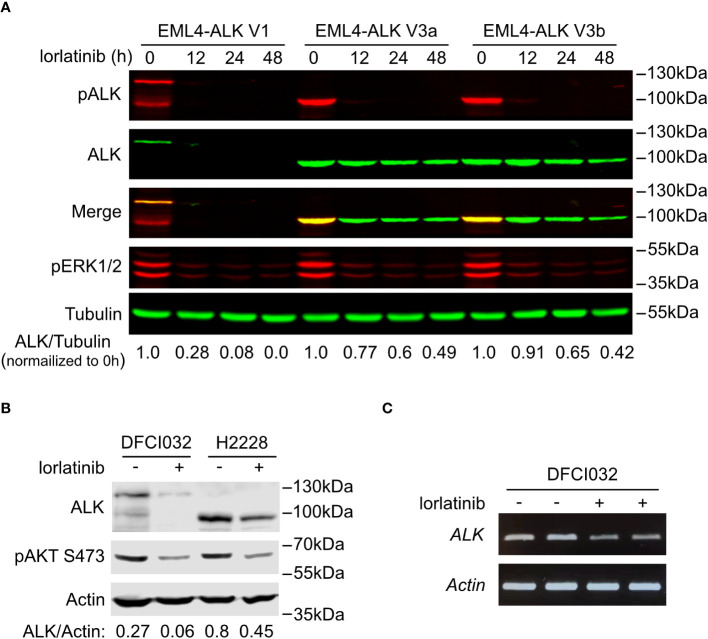
EML4-ALK V1 is more sensitive to ALK inhibition. **(A)** Time course of EML4-ALK variant inhibition with lorlatinib. PC12 cells expressing either EML4-ALK V1, V3a or V3b were treated with lorlatinib for different times as indicated, prior to immunoblotting with phospho-ALK, pan-ALK and phospho-ERK1/2 antibodies. Tubulin was used as loading control. Pan-ALK/Tubulin ratios were normalized to their respective untreated samples. **(B)** DFCI032 (harboring EML4-ALK V1) and H2228 (harboring EML4-ALK V3a/b) lung cancer cells were treated with lorlatinib for 24 hours. Whole cell lysates were analyzed by immunoblotting with pan-ALK and phospho-AKT antibodies. Actin was used as loading control. ALK/Actin ratios are indicated. **(C)** RT-PCR for *EML4-ALK V1* mRNA after ALK inhibition with lorlatinib in DFCI032 cells. *Actin* was employed as internal control.

All three constructs (EML4-ALK V1, EML4-ALK V3a and EML4-ALK V3b) expressed EML4-ALK protein of the expected size, although the level of expression of EML4-ALK V1 was lower than that of EML4-ALK V3a or EML4-ALK V3b (**Figure 1A**). The lower level of EML4-ALK V1 protein is in agreement with its previously reported less stable nature (25, 26). Accordingly, the overall phosphorylation level of EML4-ALK V1, as measured by pALK-Y1604, was lower when compared to EML4-ALK V3a or EML4-ALK V3b (**Figure 1A**). Similar results were observed in other cell lines ectopically expressing EML4-ALK variants (**Figures 2B**, **3A**) and in EML4-ALK driven lung cancer cells (**Figure 3B**). A weaker and smaller band of EML4-ALK V1 protein that can be detected by both anti-ALK and anti-pALK (Y1604) mAbs might be a truncated V1 protein lacking part of the EML4 portion. ALK is known to activate multiple downstream signaling pathways (2), including the RAS-MAPK pathway that can induce neuronal differentiation in PC12 cells (27, 28). We observed robust neurite outgrowth in PC12 cells transfected with each of the EML4-ALK variants compared with controls (**Figure 1B**). In agreement with the reduced levels of phosphorylated ALK and pERK1/2 in cells expressing the EML-ALK V1 variant when compared with EML4-ALK V3 variants (**Figure 1A**), the percentage of neurite-carrying PC12 cells expressing EML4-ALK V1 was significantly lower than that observed on expression of either EML4-ALK V3a or EML4-ALK V3b (**Figure 1C**). Taken together, these data confirm that the EML4-ALK V3a and V3b variants exhibit increased stability resulting in higher signaling output relative to EML4-ALK V1.

### 3.2 F1174S mutation impairs the kinase activity of EML4-ALK V1 and reduces its oncogenic ability

**Figure 4:**
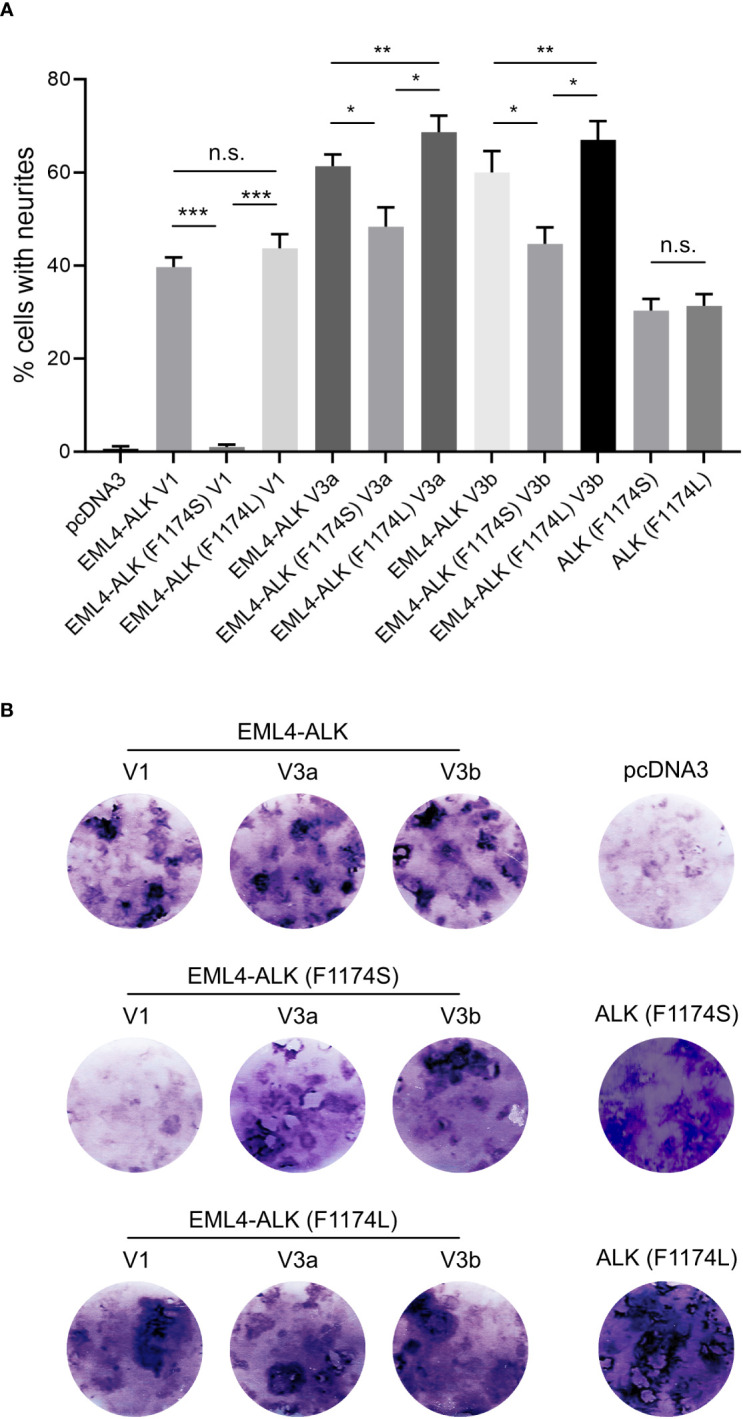
EML4-ALK (F1174S) V1 exhibits reduced ability to induce PC12 differentiation and transform NIH 3T3 cells. **(A)** Neurite outgrowth assay in PC12 cells transfected with EML4-ALK variants as indicated. Empty pcDNA3 vector, full-length ALK (F1174S) and ALK (F1174L) were employed as controls. Significance between different groups is indicated with asterisks (*p<0.05, **p<0.01, ***p<0.005, n.s. no significance, two-tailed paired student’s *t*-test). Bars represent mean percentage ± STD from three independent experiments. **(B)** Focus formation assay with NIH 3T3 cells transfected with EML4-ALK and full-length ALK variants as indicated. Images represent one set of three independent experiments. Empty pcDNA3 vector, full-length ALK (F1174S) and ALK (F1174L) were employed as controls.

To characterize the novel F1174S mutation in the context of ALK-positive NSCLC, we introduced it into EML4-ALK V1, EML4-ALK V3a and EML4-ALK V3b constructs, hereafter referred to as EML4-ALK (F1174S) V1, EML4-ALK (F1174S) V3a and EML4-ALK (F1174S) V3b. Wildtype and F1174S mutant EML4-ALK variants were expressed in PC12 cells for initial characterization. Surprisingly, we observed that EML4-ALK (F1174S) V1, EML4-ALK (F1174S) V3a and EML4-ALK (F1174S) V3b consistently exhibited reduced levels of ALK autophosphorylation compared to their wildtype counterparts, as measured by anti-pALK Y1604 levels (**Figure 2A**). Remarkably, EML4-ALK (F1174S) V1 protein levels were greatly decreased and phosphorylation of Y1604 was barely detectable (**Figures 2A, B**). In contrast, EML4-ALK (F1174S) V3a and EML4-ALK (F1174S) V3b were expressed at similar protein levels in PC12 cells (**Figure 2A**) or slightly decreased (around 20% less) protein levels in SK-N-AS cells (**Figure 2B**) compared to their wildtype counterparts. Further, EML4-ALK (F1174S) V1 only very weakly activated downstream targets such as ERK1/2 (**Figure 2A**), resulting in barely detectable neurite outgrowth (**Figure 4A**). One possible explanation is that the F1174S mutation might further destabilize the EML4-ALK V1 fusion protein, therefore leading to a marked decrease in protein levels and impaired ALK activity. Interestingly, although the F1174S mutation also affected the autophosphorylation of ALK in EML4-ALK (F1174S) V3a and EML4-ALK (F1174S) V3b, it did not affect activation of downstream targets, such as AKT and ERK significantly (**Figures 2A, B**). As a result, EML4-ALK (F1174S) V3a and EML4-ALK (F1174S) V3b were still capable of driving neurite outgrowth although at reduced levels when compared with their wildtype EML4-ALK counterparts (**Figure 4A**).

The different effect of F1174S mutation depending on the EML4-ALK fusion protein context led us to investigate further. Previous reports have shown that both F1174S and F1174L mutations are activating mutations in the context of full length ALK and are able to potentiate NB tumorigenesis together with MYCN (4, 5, 10, 29, 30). Therefore, we investigated the effect of F1174L mutation in the context of EML4-ALK fusion variants. To do this, we generated F1174L mutant EML4-ALK variants [referred to as EML4-ALK (F1174L) V1, EML4-ALK (F1174L) V3a and EML4-ALK (F1174L) V3b respectively]. These constructs were first expressed and characterized in SK-N-AS NB cells, together with the corresponding wildtype and F1174S mutant EML4-ALK counterparts. In agreement with our previous results in PC12 cells, we observed weaker autophosphorylation of EML4-ALK (F1174S) V3a and EML4-ALK (F1174S) V3b, as well as nearly undetectable autophosphorylation of EML4-ALK (F1174S) V1 (**Figure 2B**). This was in contrast to F1174L mutant EML4-ALK variants, which did not show any obvious difference from wildtype EML4-ALK variants, comparing either protein expression levels, autophosphorylation or activation of downstream targets, such as AKT and ERK in SK-N-AS cells (**Figure 2B**). All three F1174L mutant EML4-ALK variants gave slightly higher levels of neurite outgrowth in PC12 cells compared to wildtype EML4-ALK variants (**Figure 4A**). As positive controls, full length ALK harboring either F1174S or F1174L mutation [referred to as ALK (F1174S) and ALK (F1174L)] were able to induce robust differentiation of PC12 cells, consistent with previously published data (**Figure 4A**) (10). To confirm our findings further, we performed a focus formation assay in NIH 3T3 cells, which provides a straightforward test of oncogenic transforming potential (22, 31). In line with our neurite outgrowth assay in PC12 cells, EML4-ALK (F1174S) V1 was not able to transform NIH 3T3 cells and thus no obvious foci were formed, similar to vector control. All other EML4-ALK variants, either wildtype or mutant, were able to transform NIH 3T3 cells, leading to efficient focus formation (**Figure 4B**).

Finally, to exclude any possible artifact introduced during the plasmid construction, we reverted the EML4-ALK (F1174S) V1 construct to wild type [named EML4-ALK (F1174S→WT) V1], and also created the F1174S mutation from EML4-ALK (F1174L) V1 [named EML4-ALK (F1174L→S) V1]. These new constructs were expressed in PC12 cells, and as expected from our results above, EML4-ALK (F1174S→WT) V1 protein levels were recovered while those of EML4-ALK (F1174L→S) V1 decreased when compared to wildtype controls (**Supplementary Figure 1**). Taken together, these data confirm that the observed protein instability and lack of activity phenotypes are caused by the F1174S mutation, which dramatically reduces the oncogenic potential of EML4-ALK (F1174S) V1 *in vitro*.

### 3.3 EML4-ALK V1 is more sensitive to ALK inhibition

Interestingly, treatment of EML4-ALK transfected cells with the ALK inhibitor lorlatinib resulted in a decrease of EML4-ALK V1 protein levels. A similar, but much weaker effect of lorlatinib was also noted on EML4-ALK V3a and V3b variant levels (**Figure 2A**). To confirm this observation, we performed a time course of lorlatinib inhibition on EML4-ALK V1, EML4-ALK V3a or EML4-ALK V3b transfected PC12 cells. Inhibition of ALK kinase activity was indicated by phosphorylation of ALK and downstream ERK (**Figure 3A**). After twelve hours of treatment, EML4-ALK V1 protein levels were reduced to 28% compared with untreated control and almost no detectable protein was observed after twenty-four hours of lorlatinib treatment. In contrast to the rapid degradation of EML4-ALK V1, approximately 50% of EML4-ALK V3a and EML4-ALK V3b protein remained even after forty-eight hours of lorlatinib treatment (**Figure 3A**). This dependence of EML4-ALK protein stability on activity was also observed in EML4-ALK driven lung cancer cell lines. The DFCI032 NSCLC cell line expresses EML4-ALK variant 1, while the H2228 cell line contains EML4-ALK variant 3a/b. After twenty-four hours treatment with lorlatinib, endogenous EML4-ALK V1 and EML4-ALK V3a/b protein levels were measured and compared to untreated samples. Only around 20% of EML4-ALK V1 protein remained, while around 50% remained for EML4-ALK V3a/b (**Figure 3B**). When normalized to the housekeeping protein β-Actin, EML4-ALK V3a/b exhibited almost three-fold expression levels relative to EML4-ALK V1 (**Figure 3B**), in agreement with our observations in ectopically expressed samples. It is known that EML4-ALK V1 has decreased stability (25, 26, 32), but how its kinase activity supports protein levels is unclear. One hypothesis for future investigation is that ALK activity may regulate EML4-ALK V1 stability through regulation of heat shock protein 90 (Hsp90), the chaperone protein critical for EML4-ALK V1 protein stability (32, 33). Another possibility might be that ALK activity regulates the mRNA level of EML4-ALK V1. To test this possibility, we extracted total RNA from both treated and untreated DFCI032 cells and then performed RT-PCR. As expected, we observed decreased *ALK* mRNA levels in response to lorlatinib treatment (**Figure 3C**). Taken together, both protein and mRNA levels of EML4-ALK V1 are dependent on ALK signaling activity, which makes it more sensitive to ALK inhibition.

### 3.4 F1174S, but not F1174L mutation destabilizes EML4-ALK V1

It has been shown that EML4-ALK V1 has a reduced stability compared to EML4-ALK V3a and V3b due to structural defects (32). Introduction of F1174S into EML4-ALK V1, further decreased EML4-ALK V1 protein levels (**Figures 2A, B**). One possibility is that F1174S mutation may further destabilize EML4-ALK V1. To test this hypothesis, we treated cells ectopically expressing wildtype, F1174S or F1174L mutant EML4-ALK V1, EML4-ALK V3a, and EML4-ALK V3b respectively with CHX, which blocks protein synthesis.

**Figure 5:**
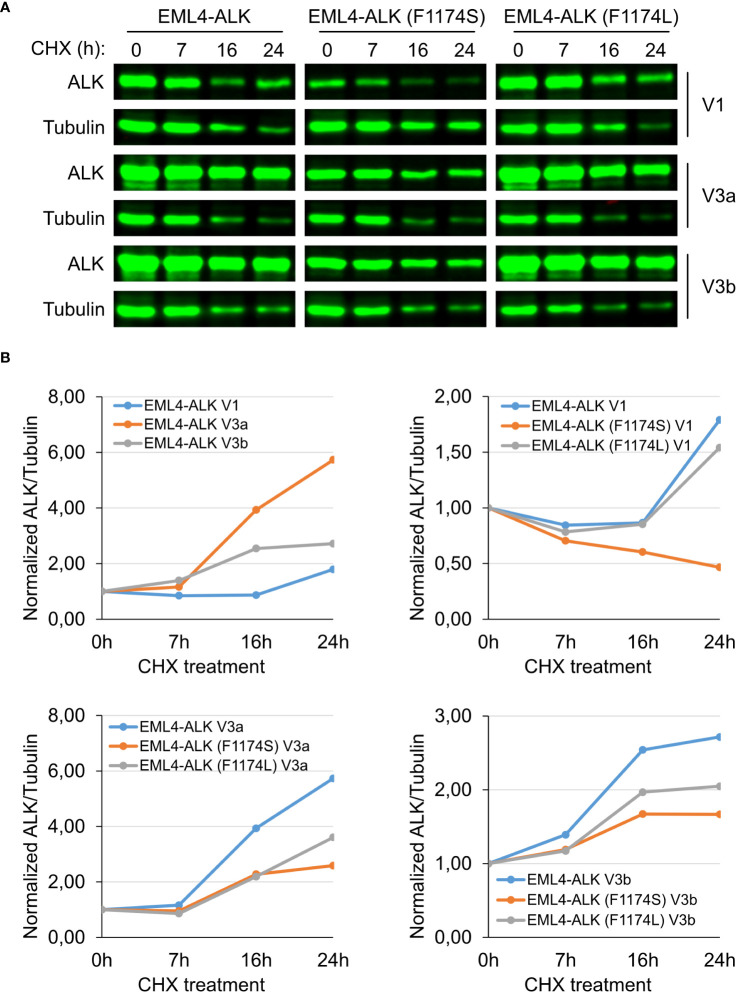
F1174S mutation destabilizes EML4-ALK (F1174S) V1 protein. **(A)** Time course of cycloheximide (CHX) treatment. NL20 cells ectopically expressing wildtype or mutant EML4-ALK variants as indicated were treated with CHX for 0, 7, 16 and 24 hours as indicated. Immunoblotting with pan-ALK was employed to detect the respective proteins. Tubulin was employed as loading control. Images representative one set of two independent experiments. **(B)** EML4-ALK fusion protein degradation dynamics. Normalized ALK/Tubulin ratios relative to untreated controls indicate the effect of F1174 mutation on EML4-ALK V1, V3a and V3b fusion protein degradation dynamics.

Firstly, when expressed in NL20 cells, EML4-ALK V1 protein levels were lower than that of either EML4-ALK V3a or V3b (**Figure 5A**), in agreement with our observations in PC12 and SK-N-AS cells (**Figures 2A, B**, **3A**). The F1174L mutation did not decrease protein expression levels of either EML4-ALK (F1174L) V1, EML4-ALK (F1174L) V3a or EML4-ALK (F1174L) V3b when compared to wildtype EML4-ALK proteins (**Figure 5A**), consistent with our observations in SK-N-AS cells (**Figure 2B**). However, in contrast to F1174L mutation, F1174S mutation greatly decreased the protein levels of EML4-ALK (F1174S) V1 to around one third of wildtype EML4-ALK V1. Protein expression levels of both EML4-ALK (F1174S) V3a and EML4-ALK (F1174S) V3b were also decreased. Secondly, after twenty-four hours CHX treatment EML4-ALK V3a and EML4-ALK V3b were protected from protein degradation, in contrast to EML4-ALK V1 that was rapidly degraded (**Figure 5A**). Normalization of ALK/β-Tubulin ratios to untreated controls, suggests that both EML4-ALK V3a and EML4-ALK V3b are rather stable proteins compared to EML4-ALK V1 (**Figure 5B**). Both EML4-ALK (F1174L) V3a and EML4-ALK (F1174L) V3b proteins displayed a slightly reduced but similar stability to EML4-ALK V3a and EML4-ALK V3b (**Figures 5A, B**), while EML4-ALK (F1174L) V1 protein was degraded in a manner similar to EML4-ALK V1, indicating that the F1174L mutation itself did not alter protein stability much. In contrast, EML4-ALK variants harboring F1174S mutation, especially EML4-ALK (F1174S) V1, exhibited significantly decreased half-lives. While both mutations affected the protein degradation of EML4-ALK V3a and V3b, the effect was more significant in V3b than V3a although they all followed a similar trend (**Figure 5B**). Taken together, our data demonstrate that the F1174S mutation further destabilizes EML4-ALK V1, leading to increased degradation, loss of kinase activity and oncogenic ability.

### 3.5 F1174S mutation sensitizes EML4-ALK V3a and V3b to crizotinib

**Figure 6:**
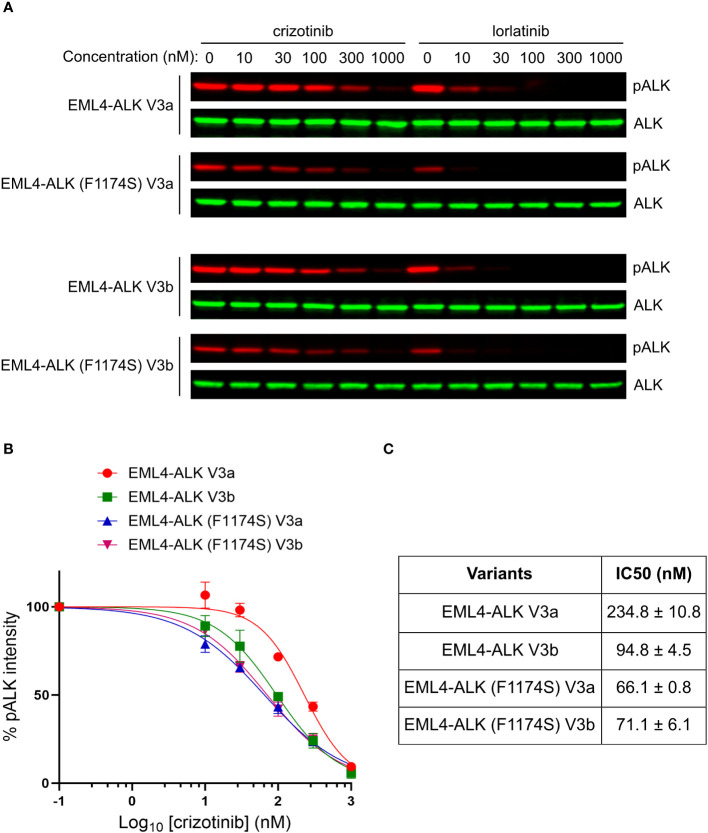
F1174S mutation sensitizes EML4-ALK V3a and V3b to crizotinib. **(A)** NL20 cells transfected with wildtype and F1174S mutant EML4-ALK V3a/b variants as indicated were treated with increased concentrations of either crizotinib or lorlatinib for 2 hours respectively. Lysates were immunoblotted with phospho-ALK and pan-ALK. **(B)** Inhibition curves of crizotinib on the four EML4-ALK proteins based on normalized pALK/ALK ratios. **(C)** IC50 of phosphorylation inhibition by crizotinib. Values represent mean ± STD from three independent experiments.

Despite the impaired autophosphorylation of EML4-ALK (F1174S) V3a and EML4-ALK (F1174S) V3b, they were still able to activate downstream signaling sufficiently and both fusion variants retained their oncogenic potential. We therefore tested the sensitivity of these EML4-ALK mutants to ALK inhibitors. To do this we expressed both wildtype and F1174S mutant EML4-ALK V3a and EML4-ALK V3b in NL20 cells respectively, prior to treatment with increasing concentrations of crizotinib or lorlatinib (from 0 nM to 1000 nM). Dose-dependent ALK activity responses were followed with anti-pALK-Y1604 as readout. Weaker ALK-Y1604 phosphorylation was observed in EML4-ALK (F1174S) V3a and EML4-ALK (F1174S) V3b expressing NL20 cells. As expected, in contrast to the requirement of high concentrations of crizotinib required for efficient inhibition, lorlatinib exhibited a superior inhibition effect (**Figure 6A**).

Based on our immunoblotting results, we generated inhibition curves (**Figure 6B**) and calculated IC50 values (concentration for 50% inhibition of ALK phosphorylation, **Figure 6C**). The results showed that, in contrast to the reported F1174C/L/V mutations, F1174S mutation indeed sensitized EML4-ALK V3a/b to crizotinib, as indicated by their IC50 values [234.8 ± 10.8 nM for EML4-ALK V3a, 94.8 ± 4.5 nM for EML4-ALK V3b, 66.1 ± 0.8 nM for EML4-ALK (F1174S) V3a and 71.1 ± 6.1 nM for EML4-ALK (F1174S) V3b]. Our results indicate that crizotinib effectively inhibits EML4-ALK (F1174S) V3a and EML4-ALK (F1174S) V3b. It is also interesting that EML4-ALK V3a and EML4-ALK V3b showed differential sensitivity to crizotinib. A recent publication shows that in H2228 cells treated with ALK inhibitors, the EML4-ALK V3a to EML4-ALK V3b ratio increased over time (21). The differential sensitivity to crizotinib may explain this observation, because the EML4-ALK V3b-containing cells are more sensitive to ALK inhibition than EML4-ALK V3a-containing ones during the treatment. An extended study showed that EML4-ALK (F1174S) V3a was also more sensitive to ALK inhibition with alectinib, brigatinib or ceritinib than EML4-ALK V3a (**Supplementary Figure 2**). Taken together, the F1174S mutation sensitizes both EML4-ALK V3a and EML4-ALK V3b to crizotinib, information that may be clinically useful.

## 4 Discussion

Since the identification of ALK fusions as oncogenic driver for 3-7% NSCLCs, numerous studies have been conducted on pathology, signaling, drug sensitivity, treatment, and resistance mechanisms of EML4-ALK in NSCLCs (11–13, 16, 19, 20, 34–40). In alignment with previous publications (25, 26, 32, 41, 42), ectopically expressed EML4-ALK variant 1 in the three different cell lines employed in this study consistently exhibited decreased protein levels and a shorter half-life time upon treatment with CHX than EML4-ALK variants 3a and 3b. We also show that protein levels of all three EML4-ALK variants investigated here decreased over time upon ALK inhibition, where the EML4-ALK variant 1 showed a faster degradation than variants 3a/b. The better progression-free survival and improved overall response rate observed in EML4-ALK V1 versus EML4-ALK V3 NSCLC patients in response to ALK TKIs treatment is in keeping with these findings (20, 43). Further, retrospective studies report that EML4-ALK V3 positive NSCLC patients have more metastatic sites and decreased progression-free survival when compared with other EML4-ALK variants (44). How ALK activity impacts on EML4-ALK variant protein levels is unclear, although interactions with proteins such as the HSP90 chaperone that is critical for protecting proteins from degradation as well as maintaining the activity and stability of oncoproteins in cancers may be of importance (33, 45). Previous reports have shown that EML4-ALK variant 1 is more dependent on HSP90 and therefore more sensitive to HSP90 inhibitors (32). The higher sensitivity of fusion protein levels to ALK inhibition may partially account for the better prognosis of patients with EML4-ALK variant 1 treated with ALK inhibitors (26). Our results also demonstrate that ALK signaling output can regulate mRNA levels of EML4-ALK. Interestingly, the promoter of EML4 harbors several putative transcription factor binding sites for STAT3, Myc and ETV5, which may regulate EML4-ALK transcription. Whether this is of significance in NSCLC will require further investigation.

**Figure 7:**
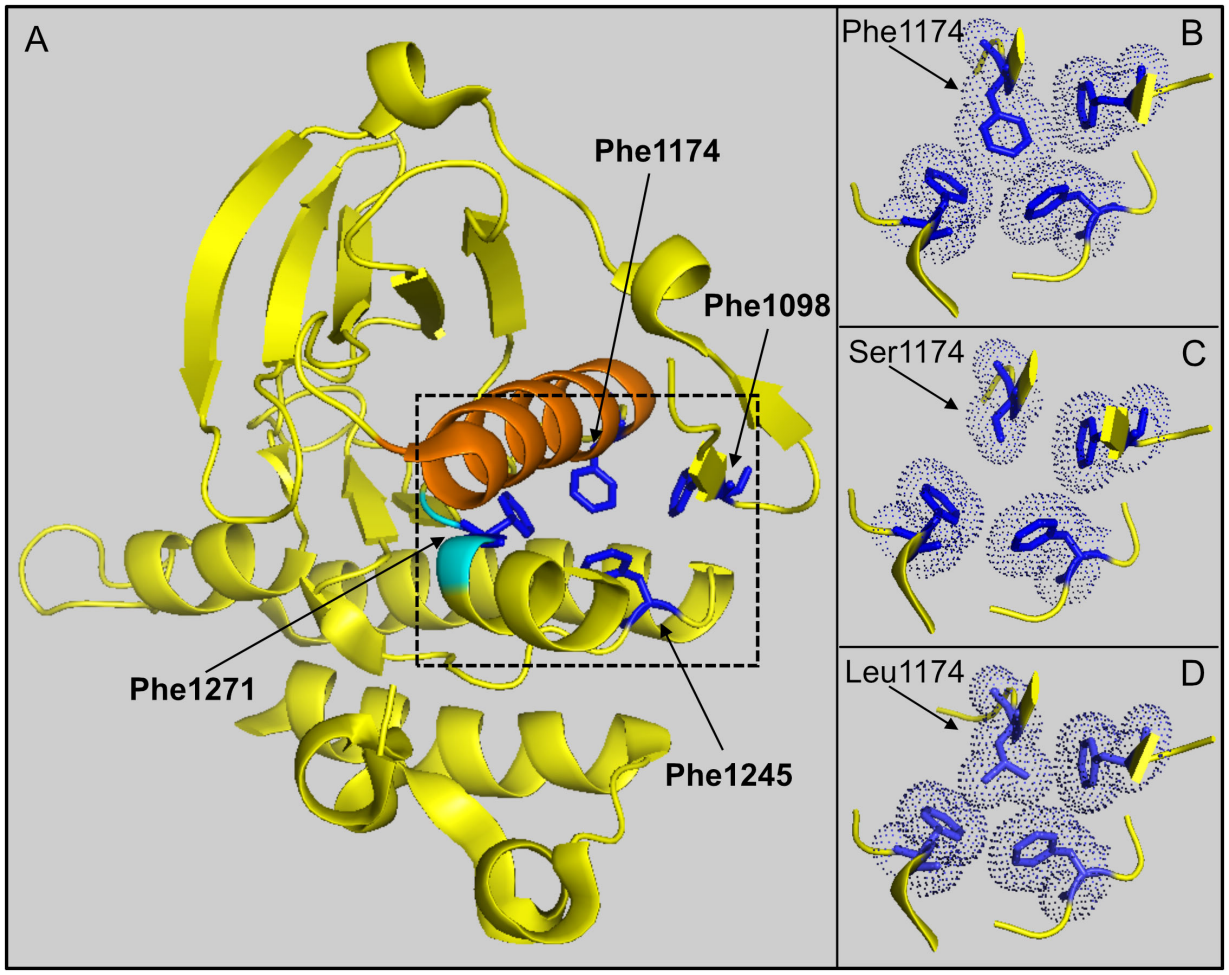
Modeling of F1174 mutations in the ALK kinase domain. **(A)** Structure depicting part of the ALK kinase domain. αC helix: orange; DFG motif: cyan. Residues F1097 (Phe1097), F1174 (Phe1174), F1245 (Phe1245) and F1271 (Phe1271) are shown in blue and indicated with arrows respectively. Image was generated with PyMOL (DeLano Scientific, Palo Alto, CA, USA) using PDB: 4CLI. **(B)** A close-up view of the hydrophobic core (dashed box in A) formed between residues F1098, F1174, F1245 and F1271. This hydrophobic core is less compact when F1174 is mutated to serine **(C)**, while it is still compact when F1174 is mutated to leucine **(D)**. The selected side chains of the four phenyalanines are shown in dotted balls and sticks.

Modeling of F1174S based on published ALK kinase domain structural data (refer to PDB: 4FNZ and 4CLI) (46, 47) allows some speculative insight into the impact of this mutation in EML4-ALK fusions. Amino acid F1174 is positioned at the base of αC-helix in the ALK kinase domain, where it interacts with amino acids F1098 in the β-turn and F1271 of the DFG motif in the activation loop (**Figures 7A, B**). Together, these three phenylalanine residues interact with a fourth phenylalanine, at position F1245, forming a hydrophobic core within the ALK kinase domain. Independent structural investigations have suggested that this hydrophobic pocket, which includes F1174, is conserved in the presence of the F1174L mutation (**Figure 7D**) (47). Thus, the position of the αC-helix, the DFG domain and the relative angle between the N- and C-terminal lobes of the kinase are very similar in both ALK wild type and ALK-F1174L crystal structures (46, 47). Modeling of the F1174S mutation (**Figure 7C**), suggests that replacing F1174 with a serine may result in destabilization of this hydrophobic pocket that may affect ALK kinase domain stability and function.

Our investigation of the effect of F1174S mutation in the context of EML4-ALK began with a mutation arising in NSCLC patient treated with ALK inhibitors. Unfortunately, we do not know the identity of the EML4-ALK variant in this patient, which is a shortcoming of our study. However, our characterization of F1174S mutation has been carried out on the most commonly observed NSCLC EML4-ALK variants (1, 3a and 3b) and clearly demonstrates clinically relevant results that differ from EML4-ALK with F1174C/L/V mutations (13, 19, 34–36, 40). According to our findings, EML4-ALK V1, V3a and V3b harboring the F1174S mutation are sensitive, rather than resistant, to crizotinib highlighting the complexity of monitoring and understanding resistance mechanisms in the NSCLC tumor landscape.

## 5 Conclusions

Taken together, our results confirm earlier reports showing that the EML4-ALK V1 fusion protein is less stable and more sensitive to ALK inhibition than EML4-ALK V3a and V3b. Moreover, our findings indicate that ALK activity may be important for the maintenance of EML4-ALK V1 protein stability and expression levels. We show that the F1174S mutation in EML4-ALK positive NSCLC impairs ALK autophosphorylation in all three EML4-ALK variants studied here (V1, V3a and V3b), in contrast to the reported ALK F1174C/L/V mutations, which cause resistance to the ALK inhibitor crizotinib. Further, F1174S further destabilizes EML4-ALK V1 and leads to decreased oncogenicity of this EML4-ALK fusion protein. Remarkably, instead of conferring resistance, F1174S mutation sensitizes EML4-ALK variants 3a and 3b to crizotinib. To our knowledge, this is the first report of a F1174S mutation in EML4-ALK positive NSCLC, and our work highlights the complexity of oncogenic drivers and their resistance mechanisms in EML4-ALK positive lung cancer patients.

## Data availability statement

The original contributions presented in the study are included in the article/**Supplementary Material**. Further inquiries can be directed to the corresponding author.

## Author contributions

JG: Investigation, Methodology, Writing – review & editing, Conceptualization, Funding acquisition, Project administration, Supervision, Writing – original draft. T-PC: Writing – review & editing, Investigation, Methodology. AV: Writing – review & editing. RP: Conceptualization, Supervision, Funding acquisition, Project administration, Writing – review & editing. BH: Conceptualization, Supervision, Funding acquisition, Project administration, Writing – review & editing.
