# Supplementary material for: ALK F1174S mutation impairs ALK kinase activity in EML4-ALK variant 1 and sensitizes EML4-ALK variant 3 to crizotinib
Source: Front Oncol. 2024 Jan 9;13:1281510. doi: 10.3389/fonc.2023.1281510 (PMC10803553; doi:10.3389/fonc.2023.1281510)

**Supplementary Data**


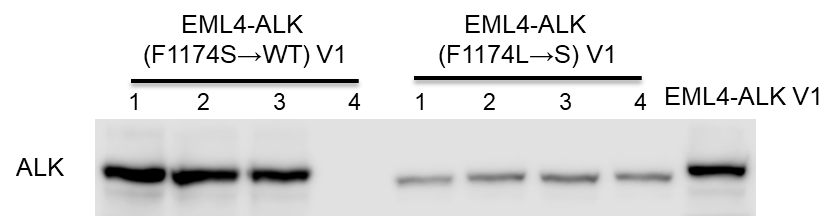


**Figure S1. Validation of the effect of F1174S mutation on the stability of EML4-ALK V1 protein.**

To exclude the possibility of artifacts introduced during the plasmid construction, the EML4-ALK (F1174S) V1 construct was mutated back to wildtype [named EML4-ALK (F1174S→WT) V1], and the F1174S mutation was generated from the EML4-ALK (F1174L) V1 construct [named EML4-ALK (F1174L→S) V1] using site-directed mutagenesis. Four colonies of each new constructs were picked, and plasmids were extracted and expressed in PC12 cells. In agreement with our observation, EML4-ALK (F1174S→WT) V1 protein levels were recovered while those of EML4-ALK (F1174L→S) V1 decreased when compared to wildtype EML4-ALK V1 controls. Note, the fourth EML4-ALK (F1174S→WT) V1 clone was not expressed for an unknown reason. All other plasmids were subsequently sequenced to confirm the correct mutagenesis.


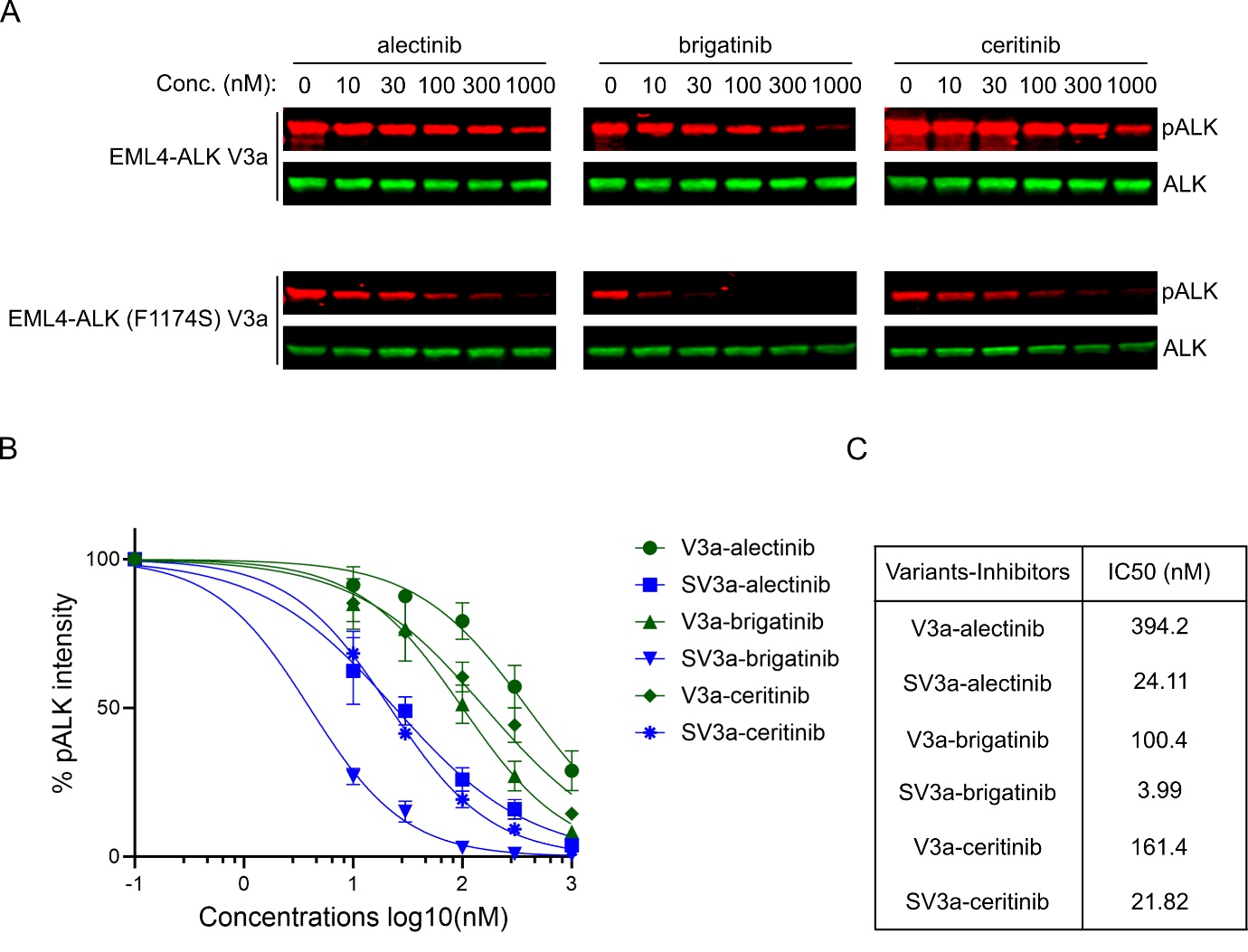


**Figure S2. F1174S mutation sensitizes EML4-ALK V3a to ALK inhibition with either alectinib, brigatinib or ceritinib.**

To test whether F1174S mutation affects the sensitivity of EML4-ALK V3 protein to alectinib, brigatinib and ceritinib ALK TKIs, we expressed both wildtype and F1174S mutant EML4-ALK V3a (denoted SV3a) in NL20 cells respectively, prior to treatment with increasing concentrations of either alectinib, brigatinib or ceritinib (from 0 nM to 1000 nM). Dose-dependent ALK activity responses were followed employing anti-pALK-Y1604 as readout. Weaker pALK-Y1604 phosphorylation was observed in EML4-ALK (F1174S) V3a expressing NL20 cells. In contrast to the high concentrations of all three tested ALK TKIs required for efficient inhibition of wildtype protein, F1174S mutation sensitized EML4-ALK V3a to all inhibitors tested, with brigatinib showing the strongest effect.

**Supplementary Data­-raw images used for review and for figures & explanation for the appearance difference**

The following several images are to show how the image processing methods were used and why the images used for peer review and for figures showed difference in appearance.


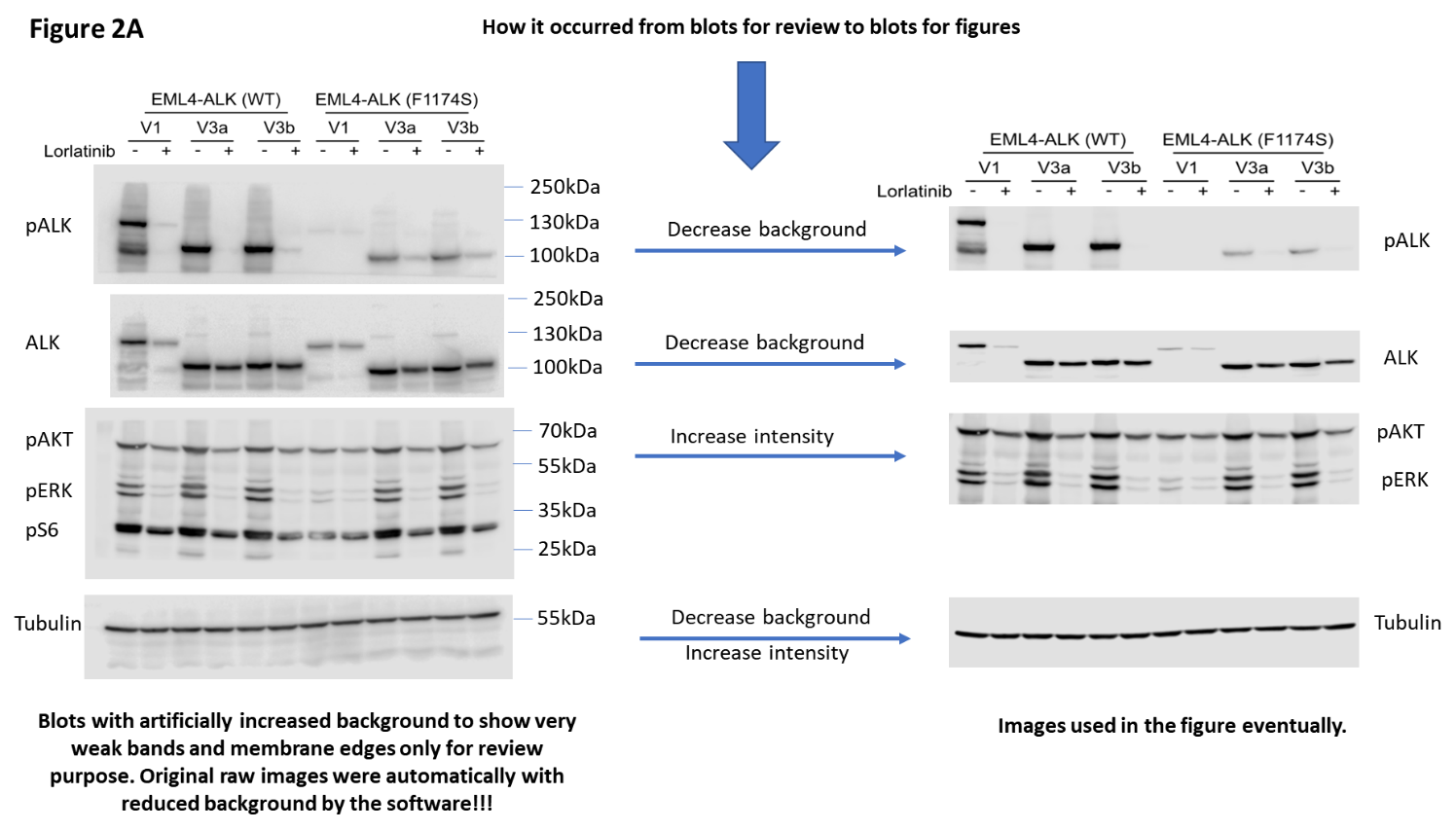


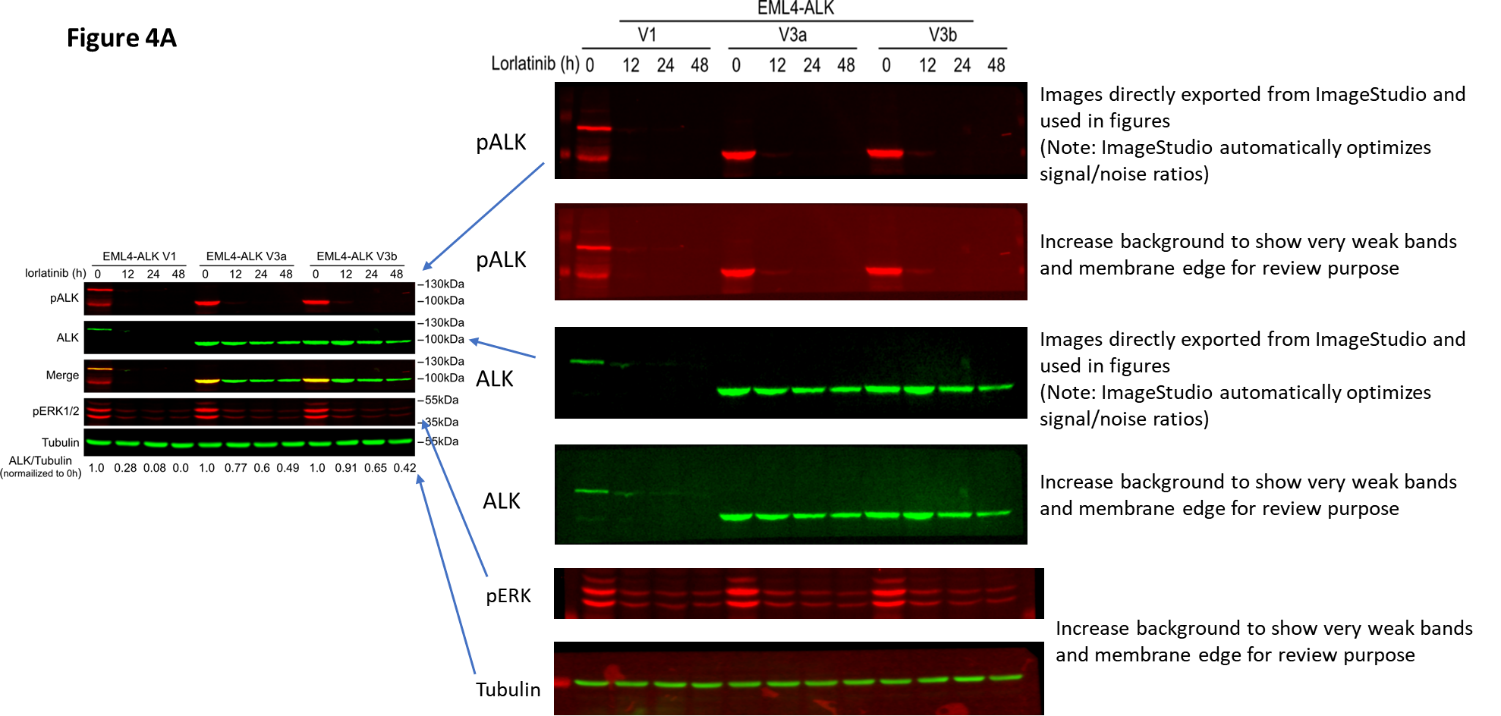


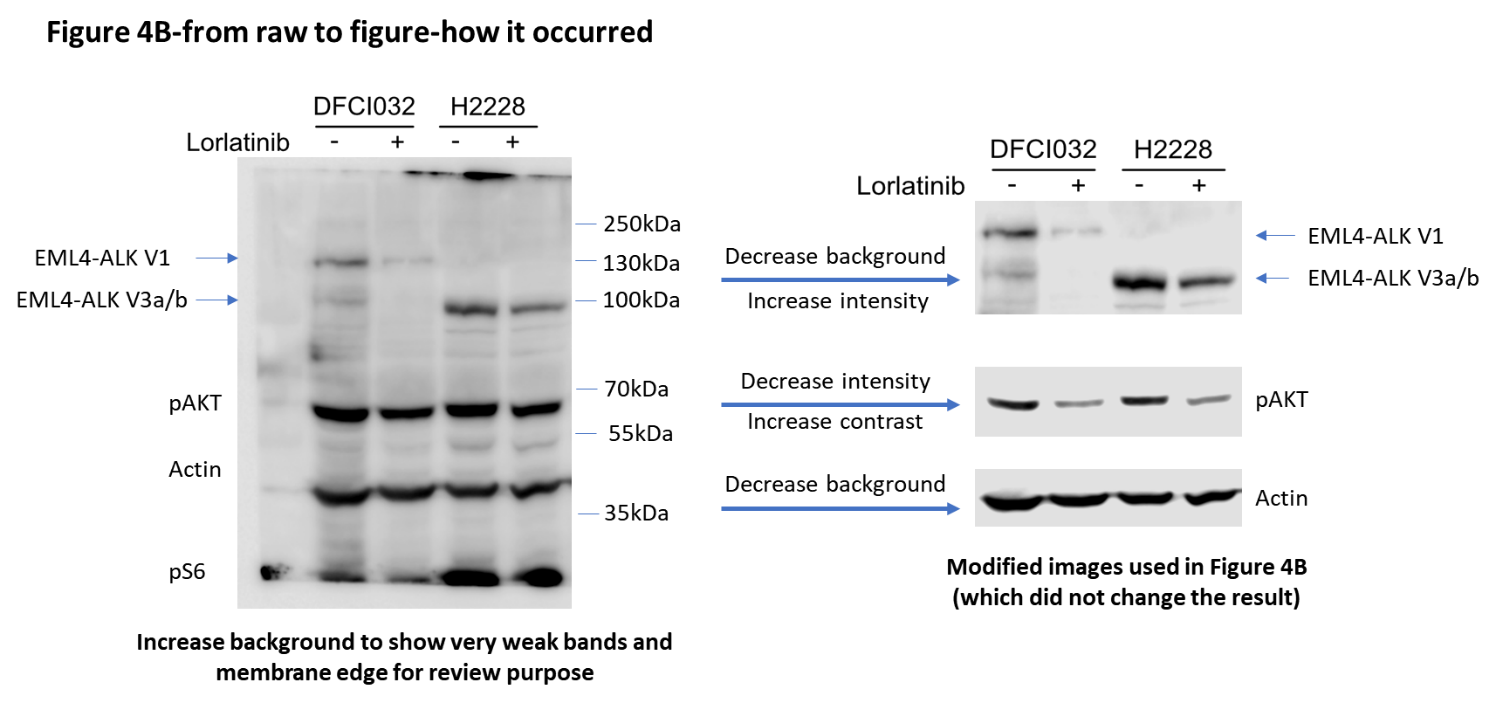


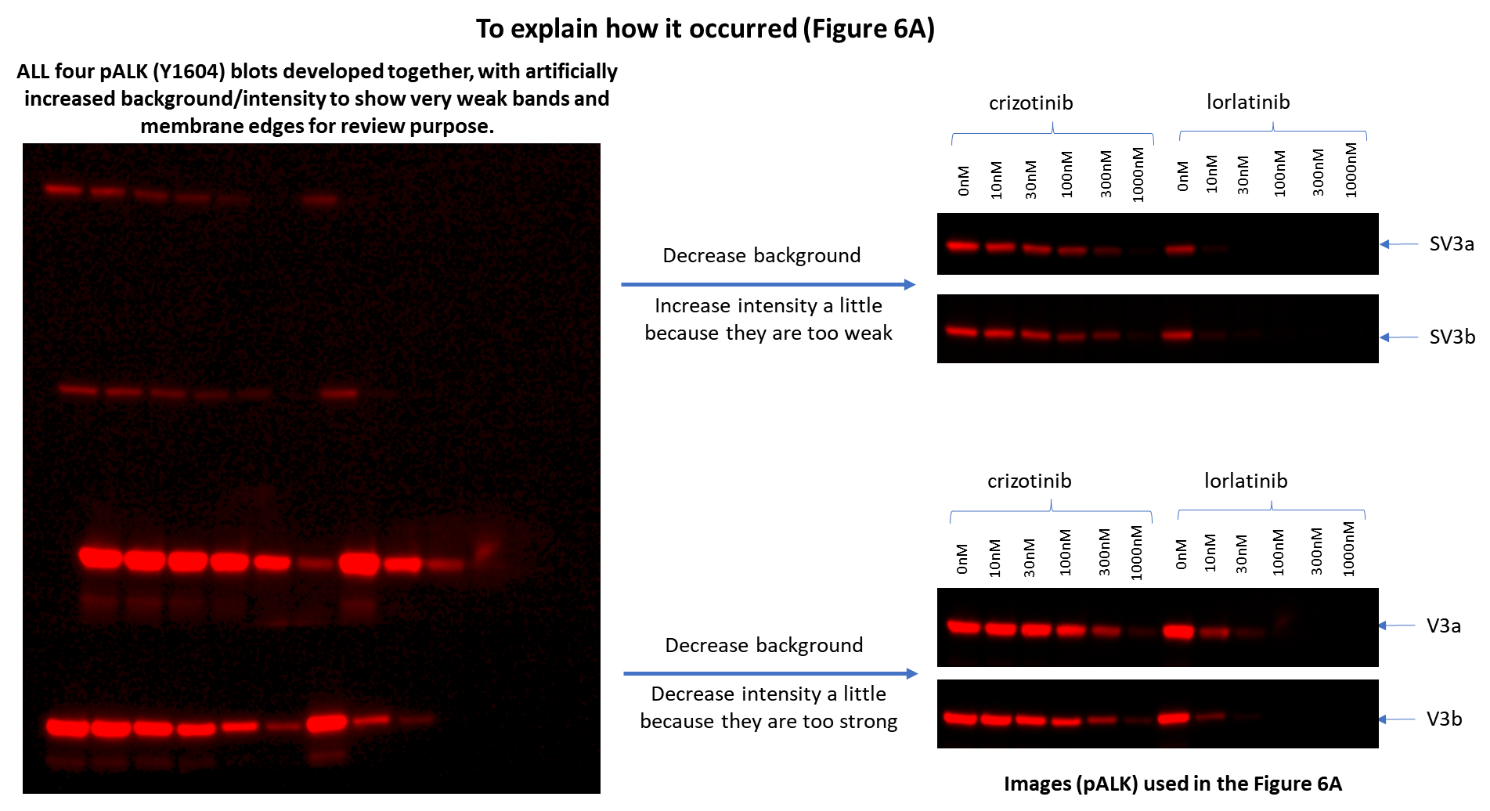

Supplement: Supplementary file 1 [file DataSheet_1.docx]
